# Supplementary material for: Development and Implementation of an Integrated Framework for Undergraduate Pharmacy Training in Maternal and Child Health at a South African University
Source: Pharmacy (Basel). 2021 Oct 8;9(4):163. doi: 10.3390/pharmacy9040163 (PMC8544740; doi:10.3390/pharmacy9040163)
Supplement: Supplementary file 1 [file pharmacy-09-00163-s001.zip › pharmacy-1340628-supplementary.pdf]

Dear B.Pharm II Student

Information sheet for participation in research

Research title: The development, and implementation of an integrated framework for undergraduate pharmacy education in maternal and child health at the University of the Western Cape.

Background

High maternal and child mortality is one of the quadruple burdens of disease facing South Africa. The reduction in mortality rate between 2009 and 2015 falls short of the targets of the Millennium Development Goals (MDGs) 4 and 5, consequently the Sustainable Development Goal (SDGs) 3. Most deaths are from preventable and curable causes.

A focus of the National Development Plan (NDP) is the reduction of maternal and child mortality. Pharmacists as part of the health workforce should be equipped with the relevant knowledge and skill required to contribute to the reduction.

Aim of study

The aim of this study is to implement a newly developed framework for an integrated training program in Maternal and Child Health (MCH) for undergraduate pharmacy students at University of the Western Cape (UWC).

Procedures

You are being asked to participate in this study because you are a 2017 B.Pharm II student at the School of Pharmacy, University of the Western Cape.

If you agree to participate in this study,

- You will complete and sign a consent form.
- You will be asked to complete a questionnaire on MCH as a baseline assessment of your knowledge and skills. The questionnaire will be returned to the investigator(s) for analysis.
- You will participate in the intervention which includes the infant growth assessment practical in the PHC 213 module, didactic lectures on contraception, pre-pregnancy and pregnancy care, infant care, communicable diseases of childhood and immunization. Lastly, service learning in Pharmacy (SLiP) at the MCH units of primary health care facilities in the Cape metropole. All of these falls under PHC 223 module.
- Subsequently, you will be asked to complete a questionnaire in a post-intervention assessment. The questionnaire will be returned to the investigator(s) for analysis.
- A 50% score in each aspect of MCH (section of the questionnaire) confers a pass mark.

The mark obtained may not contribute toward your academic achievement. It may be purely for research purpose.

Confidentiality

Your name will not be used on the questionnaire or other printed materials associated with the study. A unique ID will be allotted to each participant. Study materials will be kept in a secure location where only the senior investigators will have access to it.

Voluntary participation

Participation in this study is voluntary. Your decision to take part in the study or not will not affect your interaction with UWC and the School of Pharmacy in any way. You can choose to withdraw from the study at any time.

Risks

You would not be at risk for participating in this study.

Benefits

You will be providing important information that will help in improving curriculum content in MCH. This will ensure that pharmacy graduates from UWC SOP are competent to participate in this area of health care.

Costs

There will be no cost to you for participating in this study.

Questions

If you have any question(s), you may please speak to Mrs Elizabeth Egieyeh (0219592192, Office F12). If you are unsatisfied with the response given then please speak to anyone of the other programme co-ordinators:

Professor Angeni Bheekie: 0219592977, Office K6
Dr Mea van Huyssteen: 0219592864 Office F5
Dr Renier Coetzee: 0219593665, Office F6

UNIVERSITY of the WESTERN CAPE

School of PHARMACY

Ethics approval

The University of the Western Cape Ethics Review Board and the Registrar has granted ethics approval.

Ethics approval for the research has been obtained from University of the Western Cape Ethics Review Board, the Registrar Office and City Health (City of Cape Town).

Ethics Reference Number: HS/17/5/12
Ethics Reference Number: BM18/4/8
City Health (Western Cape): Ref: 24018

If you have any comments or concerns about participation in this study, you should first talk with the researchers. If for some reason you do not wish to do this, you may contact the University of Western Cape Ethics Review Board, which is concerned with the protection of volunteers in research projects.

Biomedical Research Ethics Committee (BMREC)
Research Development
Private Bag X17
Bellville, 7535
Tel: + 27 21 959 4111
Email: research-ethics@uwc.ac.za

UNIVERSITY of the WESTERN CAPE

School of PHARMACY

CONSENT FORM

Research title: The development, and implementation of an integrated framework for undergraduate pharmacy education in maternal and child health at the University of the Western Cape.

I confirm that I have read and understand the information sheet for the above study. I have had the opportunity to consider the information, ask questions and have had these answered satisfactorily.

1. I understand that my participation is voluntary and that I am free to withdraw at any time without giving any reason.
2. I understand that the study will not affect my legal rights and the data collected will not be linked to me in any way and full confidentiality is guaranteed
3. It has been explained clearly to me that the study was approved by relevant ethics committee
4. I agree to take part in the above study.

____________________	____________________	____________________
Participant study number	Date	Signature of participant
____________________	____________________	____________________
Name of researcher	Date	Signature of researcher

UNIVERSITY of the WESTERN CAPE

School of PHARMACY

QUESTIONNAIRE

Research title: The development, and implementation of an integrated framework for undergraduate pharmacy education in maternal and child health at the University of the Western Cape.

Unique ID:_________

A. **PERSONAL INFORMATION** (Write an X by the applicable option)

1. **Age:**
   18-30
   31-45

2. **Gender**:
   Male
   Female

3. **Parental status:**
   Children
   No Children

4. **Student's first language:**
   Afrikaans
   English
   IsiXhosa
   Other (please specify) ____

5. **Exposure to family planning, maternal and child health other than that undertaken at the School's academic program:**
   Yes
   No
   If yes, please explain ____
   ____

QUESTIONNAIRE

ALL QUESTIONS ARE ALLOTED ONE MARK EXCEPT OTHERWISE INDICATED

A. KNOWLEDGE ASSESSMENT

A1. REPRODUCTIVE/SEXUAL HEALTH

Circle the correct answer or fill the blank spaces with the correct answer as applicable.

1. Which hormones are present in combined oral contraceptives?
   a. Estrogen and progestogen
   b. Testosterone and oxytocin
   c. Prolactin and thyroxin
   d. Prolactin and oxytocin

2. Combined oral contraceptives should be started
   a. Between day 1 and day 5 of the cycle
   b. At any time in the absence of pregnancy with additional precaution (barrier method) until 7 hormonal pills have been taken
   c. After day 5 of the cycle with additional precaution (barrier method) until 7 hormonal pills have been taken
   d. All of the above

3. Which of the following contraceptive methods' effectiveness relies on the client's ability to use them correctly?
   a. Vasectomy
   b. Condoms
   c. IUD
   d. Sub-dermal implant

4. Long acting reversible contraceptives (LARC) are defined as methods that require administration once in months or cycles. Which of the methods listed below fall within this definition?
   i. Copper IUD
   ii. Levonorgestrel releasing intrauterine system
   iii. Progestogen-only injectables
   iv. Sub-dermal progestogen implants
   a. All of the above
   b. None of the above

5. Emergency contraception is absolutely indicated after sexual intercourse in all of the following situations except
   a. Slipped or broken condom
   b. Two pills forgotten during the first 7 active pills
   c. > 2 weeks late for progestogen-only injections
   d. <2 weeks late for progestogen-only injections

6. The following information is important before emergency contraception is dispensed except one?
    a. Date of the last menstrual period
    b. <120 hours since the last episode of unprotected intercourse
    c. How many hours of rest observed
    d. Exclusion of pregnancy

7. Oral progestogen-only pills are
    a. Preferred postpartum during lactation
    b. Preferred in women over 35 years who smoke and have increased risk of cardiovascular disease
    c. None of the above
    d. All of the above

8. Rifampicin, Lopinavir/Ritonavir, Nevirapine are enzyme inducers that interact with oral contraceptives to
    a. Reduce contraceptive effect
    b. Increase contraceptive effect
    c. Stabilize contraceptive effect
    d. Have no effect on the contraceptive

9. Dual contraception with a barrier method is encouraged to prevent __________________
   ____

A2. MATERNAL/ANTENATAL CARE

Circle the correct answer or fill the blank spaces with the correct answer as applicable.

1. All non-pregnant women of reproductive age should be advised to commence periconceptual folic acid supplementation (women planning pregnancy).
    a. True
    b. False

2. Every pregnant woman should have at least ____ antenatal clinic visits.
    a. 2
    b. 4
    c. 6
    d. 8

3. Which of the following lifestyle modifications are required by a pregnant woman for a healthy pregnancy and baby?
    i. no smoking
    ii. no alcohol intake
    iii. balanced diet
    iv. strenuous exercise
    a. All of the above
    b. None of the above
    c. I, ii, iii
    d. I, ii, iv

4. Which of the following are danger signs in pregnancy?
   i. nausea and vomiting
   ii. vaginal bleeding
   iii. baby not moving
   iv. severe abdominal pain

   a. All of the above
   b. None of the above
   c. i ii iii
   d. ii iii iv

5. When is ARV therapy initiated in newly diagnosed HIV positive pregnant women?
   a. Immediately HIV status is confirmed
   b. CD4 count <500 cells/mm^3
   c. Viral load <1000 IU/ml
   d. Immediately after birth

6. Which of the following is teratogenic?
   a. ACE inhibitors
   b. Vitamin A derivatives
   c. Statins
   d. A, B, C
   e.

7. Which of these factors influence the manifestation and severity of teratogenicity?
   a. Gestation period
   b. Dose and duration of therapy
   c. Degree of drug transfer across the placenta
   d. All of the above

Please answer questions 8, 9 and 10 in the table below according to the instruction at the top of the table. (4 marks)

**Complaint**	**State one cause in the blank cell.**	**Give one Non-pharmacological treatment/prevention**
8. Morning sickness	Reduced gastric motility, high hormonal levels	
9. Heartburn		
10. Vaginal thrush	Alteration in pH balance	

NEONATAL AND CHILD CARE

Circle the correct answer or fill the blank spaces with the correct answer as applicable.

1. Exclusive breastfeeding (EBF) is defined as giving only breast milk to infants for the first ______ of life.
   a. 2 months
   b. 4 months
   c. 6 months
   d. 12 months

2. An HIV exposed infant is one whose mother is HIV infected or whose HIV infection has not been confirmed or excluded. Which ARV medication is given to such infants at birth?

   ____

3. WHO recommends that HIV positive women who are on ART should exclusively breastfeed their babies?
   a. True
   b. False

4. Cracked nipples during breastfeeding is a result of
   i. poor positioning of the baby to the nipple
   ii. incorrect attachment to the nipple
   iii. removing the baby from the breast before suction is broken
   iv. breastfeeding the baby while lying down
   a. All of the above
   b. i ii iii
   c. i and iv

5. The Expanded Program on Immunization (EPI) covers the major killer diseases of infanthood. List three of such diseases. (3 marks)

   ____
   ____
   ____

6. What is the first line treatment of diarrhea in infants and children according to the standard treatment guidelines? ______________________________

   ____

7. Deworming agents are initially given to children at ____________, subsequently every ______.
   a. 6 months, 6 months
   b. 6 months, 12 months
   c. 12 months, 6 months
   d. 12 months, 12 months

8. Outline one pharmacological and non-pharmacological treatment for diaper/nappy rash (2 marks)
   Pharmacological (according to STGs):____
   Non-pharmacological:____

9. Which of the following may be used in the treatment of mastitis?
   a. Apply warm compresses
   b. Drink plenty of clear fluids
   c. Panado
   d. Antibiotics
   e. All of the above

B. SKILLS ASSESSMENT

The SLIP 213 class undertook a M&CH practical session in the first semester. Based on that exposure, please give a brief explanation to each of the following questions.

1. Please explain how an infant's (0-12 months) height is measured? (2 marks)
   ____
2. Should an infant be fully clothed or undressed during weight measurement? (1 mark)
   ____
3. How is an infant's head circumference measured? (2 marks) ____________________
   ____
4. Why is an infant's head circumference measured? (1 mark) ____________________
   ____
5. What is MUAC? (1 mark)____
   ____
6. Why is MUAC measured? (1 mark) ____
   ____

* THE END. THANK YOU!!!

FACULTY OF NATURAL SCIENCES
Private Bag X17 Bellville 7535
Telephone +27 21 9592190
Fax +27 21 9593407

APPENDIX 1

Unique ID: ______________

Dear B.Pharm IV Student

Information sheet for participation in research

Research title: The development, and implementation of an integrated framework for undergraduate pharmacy education in maternal and child health at the University of the Western Cape.

Background

High maternal and child mortality is one of the quadruple burdens of disease facing South Africa. The reduction in mortality rate between 2009 and 2015 falls short of the targets of the Millennium Development Goals (MDGs) 4 and 5, consequently the Sustainable Development Goal (SDGs) 3. Most deaths are from preventable and curable causes.

A focus of the National Development Plan (NDP) is the reduction of maternal and child mortality. Pharmacists as part of the health workforce should be equipped with the relevant knowledge and skill required to contribute to the reduction.

Aim of study

The aim of this study is to assess your MCH knowledge and skills following your exposure to the integrated framework in Maternal and Child Health (MCH) for undergraduate pharmacy students at the University of the Western Cape (UWC).

Procedures

You are being asked to participate in this study because you are a 2019 B.Pharm IV student at the School of Pharmacy, University of the Western Cape and you participated in the baseline study and intervention in your second year of study (2017 BPharm II) as well as other MCH components in the other years of study.

If you agree to participate in this study,

- You will complete and sign a consent form.
- You will be asked to complete a questionnaire on MCH as a 2019 post intervention assessment which is being carried out two years after the intervention as an assessment of your knowledge and skills. The questionnaire will be returned to the investigator(s) for analysis.
- A 50% score in each aspect of MCH (section of the questionnaire) confers a pass mark.

The mark obtained may not contribute toward your academic achievement. It may be purely for research purpose.

Confidentiality

Your name will not be used on the questionnaire or other printed materials associated with the study. A unique ID will be allotted to each participant. Study materials will be kept in a secure location where only the senior investigators will have access to it.

Voluntary participation

Participation in this study is voluntary. Your decision to take part in the study or not will not affect your interaction with UWC and the School of Pharmacy in any way. You can choose to withdraw from the study at any time.

Risks

You would not be at risk for participating in this study.

Benefits

You will be providing important information that will help in improving curriculum content in MCH. This will ensure that pharmacy graduates from UWC SOP are competent to participate in this area of health care.

Costs

There will be no cost to you for participating in this study.

Questions

If you have any question(s), you may please speak to Mrs Elizabeth Egieyeh (0219592192, Office F12). If you are unsatisfied with the response given then please speak to anyone of the other programme co-ordinators:
Professor Angeni Bheekie: 0219592977, Office K6
Dr Mea van Huyssteen: 0219592864 Office F5
Dr Renier Coetzee: 0219593665, Office F6

Ethics approval

The University of the Western Cape Ethics Review Board and the Registrar has granted ethics approval.

Ethics approval for the research has been obtained from University of the Western Cape Ethics Review Board, the Registrar Office and City Health (City of Cape Town).

Ethics Reference Number: HS/17/5/12
Ethics Reference Number: BM18/4/8
City Health (Western Cape): Ref: 24018

If you have any comments or concerns about participation in this study, you should first talk with the researchers. If for some reason you do not wish to do this, you may contact the University of Western Cape Ethics Review Board, which is concerned with the protection of volunteers in research projects.

Biomedical Research Ethics Committee (BMREC)
Research Development
Private Bag X17
Bellville, 7535
Tel: + 27 21 959 4111
Email: research-ethics@uwc.ac.za

FACULTY OF NATURAL SCIENCES
Private Bag X17 Bellville 7535
Telephone +27 21 9592190
Fax +27 21 9593407

APPENDIX 2

CONSENT FORM

Research title: The development, and implementation of an integrated framework for undergraduate pharmacy education in maternal and child health at the University of the Western Cape.

I confirm that I have read and understand the information sheet for the above study. I have had the opportunity to consider the information, ask questions and have had these answered satisfactorily.

1. I understand that my participation is voluntary and that I am free to withdraw at any time without giving any reason.
2. I understand that the study will not affect my legal rights and the data collected will not be linked to me in any way and full confidentiality is guaranteed
3. It has been explained clearly to me that the study was approved by relevant ethics committee
4. I agree to take part in the above study.

____________________	____________________	____________________
Participant Unique ID	Date	Signature of participant

____________________	____________________	____________________
Name of researcher	Date	Signature of researcher

UNIVERSITY of the WESTERN CAPE

School of PHARMACY

QUESTIONNAIRE

Research title: The development, and implementation of an integrated framework for undergraduate pharmacy education in maternal and child health at the University of the Western Cape.

Unique ID:____________________

A. PERSONAL INFORMATION/ DEMOGRAPHCS (check as required)

1. AGE:

☐ 20 - 30 years

☐ 31 – 40 years

☐ 41-50 years

2. GENDER:

☐ Male

☐ Female

3. PARENTAL STATUS:

☐ Children

☐ No children

4. LOCUMING :

☐ Yes

☐ No

IF YES, HOW LONG:

☐ 1-2 years

☐ > 3 years

ALL QUESTIONS ARE ALLOCATED ONE MARK EXCEPT OTHERWISE INDICATED

B. KNOWLEDGE SECTION:

B1. REPRODUCTIVE/SEXUAL HEALTH (Circle the correct answer or “I don’t know” if you are not sure of the correct answer. Also, fill the blank spaces with the correct answer or “I don’t know” if you are not sure of the correct answer)

1. Which hormones are present in combined oral contraceptives?
    a. Eostrogen and progestogen
    b. Testosterone and oxytocin
    c. Prolactin and thyroxin
    d. Prolactin and oxytocin
    e. I don’t know
2. Combined oral contraceptives should be started
    a. Between day 1 and day 5 of the cycle
    b. At any time in the absence of pregnancy
    c. After day 5 of the cycle with additional precaution (barrier method) until 7 hormonal pills have been taken
    d. All of the above
    e. I don’t know
3. Which of the following contraceptive methods’ effectiveness relies on the client’s ability to use them correctly?
    a. Vasectomy
    b. Condoms
    c. IUD
    d. Sub-dermal implant
    e. I don’t know
4. Long acting reversible contraceptives (LARC) are defined as methods that require administration once in months or cycles. Which of the methods listed below fall within this definition?
    i. Copper IUD
    ii. Levonorgestrel releasing intrauterine system
    iii. Progestogen-only injectables
    iv. Sub-dermal progestogen implants
        a. All of the above
        b. None of the above
        c. ii and iii
        d. i and ii
        e. I don’t know
5. Emergency contraception is absolutely indicated after sexual intercourse in all of the following situations except
    a. One pill forgotten or 3 hours late with sexual intercourse in the past 5 days
    b. Two pills forgotten during the first 7 active pills
    c. > 2 weeks late for Progestogen-only injections
    d. <2 weeks late for Progestogen-only injections

e. I don't know

6. The following information are important before emergency contraception is dispensed except one?
    a. Date of the last menstrual period
    b. <120 hours since the last episode of unprotected intercourse
    c. How many hours of rest observed
    d. Exclusion of pregnancy
    e. I don't know
7. Dual contraception is encouraged to prevent the transmission of ____________________
8. Oral progestogen-only pills are
    a. Preferred postpartum during lactation
    b. In women over 35 years who smoke and have increased risk of cardiovascular disease
    c. Disrupt the menstrual cycle
    d. All of the above
    e. I don't know
9. Rifampicin, Lopinavir/Ritonavir, Nevirapine are enzyme inducers that interact with oral contraceptives to
    a. Reduce contraceptive effect
    b. Increase contraceptive effect
    c. Stabilize contraceptive effect
    d. Terminate contraceptive effect
    e. I don't know

B2. MATERNAL/ANTENATAL CARE (Circle the correct answer or "I don't know" if you are not sure of the correct answer. Also, fill the blank spaces with the correct answer or "I don't know" if you are not sure of the correct answer)

10. All non-pregnant women of reproductive age should be advised to commence periconceptual folic acid supplementation (women planning pregnancy). True or false or I don't know?
11. Every pregnant woman should have at least ____ antenatal clinic visits.
    a. 2
    b. 4
    c. 6
    d. 8
    e. I don't know
12. State 4 lifestyle modifications required by a pregnant woman for a healthy pregnancy and baby? (4 marks)

____

____

____

____

13. State 4 danger signs of pregnancy. (4 marks)

____

____

____

14. When is ARV therapy initiated in newly diagnosed HIV positive pregnant women?
    a. Immediately HIV status is confirmed
    b. CD4 count <500 cells/mm^3
    c. Viral load <1000 IU/ml
    d. Immediately after birth
    e. I don't know
15. Which of the following is non-teratogenic?
    a. ACE inhibitors
    b. Vitamin A derivatives
    c. Statins
    d. None of the above
    e. I don't know
16. Which of these factors influence the manifestation and severity of teratogenicity?
    a. Gestation period
    b. Dose and duration of therapy
    c. Degree of drug transfer across the placenta
    d. All of the above
    e. I don't know

Please answer questions 17, 18 and 19 in the table below according to the instruction at the top of the table. (4 marks)

**Complaint**	**State one cause in the blank cell.**	**Give one Non-pharmacological treatment/prevention**
17. Morning sickness	Reduced gastric motility, high hormonal levels	
18. Heartburn		
19. Vaginal thrush	Alteration in pH balance	

B3. NEONATAL AND CHILD CARE (Circle the correct answer or "I don't know" if you are not sure of the correct answer. Also, fill the blank spaces with the correct answer or "I don't know" if you are not sure of the correct answer)

19. Exclusive breastfeeding (EBF) is defined as giving only breast milk to infants for the first _______ of life.
    a. 2 months
    b. 4 months
    c. 6 months
    d. 12 months

e. I don't know

20. An HIV exposed infant is one whose mother is HIV infected or whose HIV infection has not been confirmed or excluded. Which ARV medication is given to such infants at birth?

____

21. WHO recommends that HIV positive women who are on ART should exclusively breastfeed their babies. True or false?
22. Cracked nipples during breastfeeding is a result of
    i. Poor positioning of the baby to the nipple
    ii. Incorrect attachment to the nipple
    iii. Removing the baby from the breast before suction is broken
    iv. Breastfeeding the baby while lying down
    a. All of the above
    b. i, ii, iii
    c. i and ii
    d. Ii and iii
    e. I don't know
23. The Expanded Program on Immunization (EPI) covers the major killer diseases of infanthood. List three of such diseases. (3 marks)

____
____
____

24. How is diarrhea treated in infants and children?

____
____
____

25. Deworming agents are initially given to children at ______________, subsequently every ______.
    a. 6 months, 6 months
    b. 6 months, 12 months
    c. 12 months, 6 months
    d. 12 months, 12 months
    e. I don't know
26. Outline one pharmacological and non-pharmacological treatment ***for diaper/nappy rash*** (2 marks)

Pharmacological:____
Non-pharmcological:____
____

27. Which of the following can be used in the treatment of mastitis?
    a. Apply warm compresses
    b. Drink plenty of clear fluids
    c. Acetaminophen tablets
    d. Antibiotics
    e. All of the above
    f. I don't know

C. **SKILLS ASSESSMENT** (Please give a brief explanation to each of the following questions or "I don't know" if you are not sure of the correct answer)

28. Please explain how an infant's (0-12 months) height is measured? (2 marks)

29. Should an infant be fully clothed or undressed during weight measurement? (1 mark)

30. How is an infant's head circumference measured? (2 marks)

31. Why is an infant's head circumference measured? (1 mark)

32. What is MUAC? (1 mark)

33. Why is MUAC measured? (1 mark)

*Thank you!!
